# Occlusal disharmony accelerates the initiation of atherosclerosis in apoE knockout rats

**DOI:** 10.1186/1476-511X-13-144

**Published:** 2014-09-05

**Authors:** Daisuke Ekuni, Toshiki Yoneda, Yasumasa Endo, Kenta Kasuyama, Koichiro Irie, Shinsuke Mizutani, Tetsuji Azuma, Takaaki Tomofuji, Manabu Morita

**Affiliations:** Department of Preventive Dentistry, Okayama University Graduate School of Medicine, Dentistry and Pharmaceutical Sciences, 2-5-1 Shikata-cho, Kita-ku, Okayama, 700-8558 Japan; Department of Preventive Dentistry and Dental Public Health, Aichi-Gakuin University, Kusumoto-cho, Chikusa-ku, Nagoya, 464-8650 Japan; Advanced Research Center for Oral and Craniofacial Sciences, Okayama University Dental School, 2-5-1 Shikata-cho, Kita-ku, Okayama, 700-8558 Japan

**Keywords:** Apolipoprotein E, Atherosclerosis, Occlusion, Stress, Toll-like receptor, Vascular cell Adhesion molecule-1

## Abstract

**Background:**

Psychosocial stress is one of the risk factors for atherosclerosis. As occlusal disharmony induces psychological stress, we hypothesized that psychological stress by occlusal disharmony accelerates atherosclerosis. The aim of this study was to investigate the effects of occlusal disharmony on the initiation of atherosclerosis in apolipoprotein E (apoE) knockout rats.

**Methods:**

Fourteen male apoE-knockout rats (age; 8 weeks) (Sprague–Dawley strain background) were divided into two groups of seven rats: the occlusal disharmony group and the no treatment (control) group. In the occlusal disharmony group, the maxillary molar cusps were cut off for the 8-week experimental period.

**Results:**

In the occlusal disharmony group, the percentages of the area of total aortic lumen occupied by plaques and lipid were significantly higher than those in the control group (*p* < 0.05, *t*-test). The occlusal disharmony group also showed significantly higher serum levels of very low-density lipoprotein-cholesterol (VLDL) and low-density lipoprotein-cholesterol (LDL), plasma levels of corticosterone (1.9, 1.3 and 1.3 times, respectively), higher aortic protein expression levels of vascular cell adhesion molecule-1 (VCAM1) and intercellular adhesion molecule-1 (ICAM1) (1.5 and 1.4 times, respectively), and higher aortic gene expression of levels of *VCAM1* and *Toll-like receptor 4* (*TLR4*) (1.9 and 4.3 times, respectively), as compared to the control group (*p* < 0.05). However, there were no significant differences in serum levels of oxidized LDL, reactive oxygen metabolites and C-reactive protein between the two groups.

**Conclusion:**

In apoE knockout rats, occlusal disharmony may induce VCAM1, ICAM1 and TLR4 expression and accelerate the initiation of atherosclerosis.

## Background

Atherosclerosis is a chronic disease of the arterial wall that leads to death and loss of productive life years worldwide [1]. The pathogenesis of atherosclerosis can be divided into three phases: initiation, lesion progression and thrombotic complication [1]. The initial step in the development of atherosclerotic plaques is mediated, in part, by the monolayer of endothelial cells lining the inner wall of the arterial vessel [2]. According to various stimuli, focal areas of the endothelial monolayer are activated to express adhesion molecules, such as vascular cell adhesion molecule-1 (VCAM1) and intercellular adhesion molecule-1 (ICAM1), and capture leukocytes. After adhering to activated endothelial cells, leukocytes are recruited to enter the intima by chemoattractant signals [3]. Low-density lipoprotein-cholesterol (LDL) particles deposit in the artery wall and monocyte-derived foam cells accumulate in lesions [3]. Toll-like receptor 4 (TLR4), a critical key factor in regulating innate immune response, plays an important role in the formation of foam cells [4]. At the same time, reactive oxygen species (ROS) peroxidize lipid components, leading to the formation of oxidized LDL-cholesterol (ox-LDL), one of the key mediators of atherosclerosis [5].

Numerous risk factors contribute to atherosclerosis, including smoking [6], diabetes mellitus [7], dyslipidemia [8], hypertension [9, 10] and periodontitis [11, 12]. However, the absence of such traditional risk factors does not completely protect from the disease, which indicates additional factors involved in the development of atherosclerosis [13]. Evidence reveals that a chronic inflammatory response contributes to atherogenesis and plaque disruption, and the innate immune response plays a critical role in the initiation of this process [14, 15]. Oxidative stress is also an important manifestation of the inflammatory responses during the atherogenesis [16].

Psychosocial stress, particularly chronic stress, is one of these nontraditional risk factors for atherosclerosis in humans [17, 18]. Experimental studies also demonstrated that chronic stress accelerates atherosclerosis [19, 20]. For example, chronic unpredictable stress accelerates atherosclerosis in apolipoprotein E (apoE) knockout mice [20]. However, the mechanisms leading to atherosclerosis initiation are not fully understood.

Occlusal disharmony, which includes malocclusion with reduced masticatory performance, induces psychological stress. In humans, malocclusion may contribute to psychological stress in young adults [21]. In animal models, the placement of acrylic caps on both lower incisors in rats increases plasma corticosterone levels, an indicator of psychological stress [22]. The molarless state in aged senescence-accelerated prone mice induces higher plasma corticosterone levels when compared to molar-intact control mice [23], and cutting off maxillary molar crowns increases plasma corticosterone levels in both wild-type and apoE knockout rats [24]. However, it is unclear whether psychological stress induced by occlusal disharmony is able to accelerate atherosclerosis.

We hypothesized that psychological stress induced by occlusal disharmony accelerates atherosclerosis. The aim of this study was to investigate the effects of occlusal disharmony on the initiation of atherosclerosis in apoE knockout rats as an established atherosclerosis model [20]. The main outcomes of the present study were the percentages of the area of total aortic lumen occupied by plaques and the area of lipid deposition. To clarify the mechanisms of initiation of atherosclerosis, aortic expression of VCAM1, and TLR4 and serum lipid profiles were evaluated. In addition, plasma corticosterone levels were determined as a parameter of psychological stress by occlusal disharmony [24]. Furthermore, levels of reactive oxygen metabolites (ROM) (whole oxidant capacity of serum against N, N-diethylparaphenylendiamine in acidic buffer) were determined as a marker of circulating ROS levels [25], and levels of C-reactive protein (CRP) were determined as a marker of inflammation [20].

## Materials and methods

### Animals

Fourteen male apoE-deficient rats (age, 8 weeks) (Sprague–Dawley background) were obtained from Sigma Laboratory (St. Louis, MO) for this 8-week study. Animals were housed in an air-conditioned room (23-25°C) with a 12-h light–dark cycle. Animals had free access to powdered diet (Oriental Yeast Co., Tokyo, Japan) and tap water. The experimental protocol was approved by the Animal Care and Use Committee of Okayama University (OKU-2012428).

### Experimental design

Rats were randomly divided into two groups of seven rats each: a control group, which received no treatment for 8 weeks, and an occlusal disharmony group, in which all maxillary molar crowns were cut off at the gingival margin using a dental turbine for 8 weeks [26]. All procedures were performed under general anesthesia by inhalation of 2-4% isoflurane delivered in an O_2_ gas. Animals were sacrificed under general anesthesia after 8 weeks.

### Measurement of plasma corticosterone, and serum total cholesterol, triglyceride, lipoproteins, ox-LDL, ROM and CRP levels

Plasma samples were collected from the heart at 8 weeks, between 7:00 am and 9:00 am, in tubes containing EDTA (Terumo, Tokyo, Japan) [27]. Tubes were immediately placed on ice and then centrifuged at 2,000 × *g* for 10 min at 4°C. Supernatants were collected and stored at −80°C before use. Plasma corticosterone levels were determined using an enzyme immunoassay kit (Yanaihara Institute Inc., Shizuoka, Japan) in accordance with the manufacturer’s instructions.

Serum samples were separated by centrifugation at 1,500 × *g* for 15 min. In addition to serum total cholesterol and triglyceride, serum lipoproteins were analyzed by a gel permeation high performance liquid chromatography system at Skylight Biotech (Akita, Japan), as described previously [28]. We quantified individual subfractions using best curve fitting analysis, assuming that the particle sizes for all subfractions followed a Gaussian distribution. Particle sizes for individual subfractions were determined as 30–80 nm (very low-density lipoprotein-cholesterol [VLDL]), 16–30 nm (LDL) and 8–16 nm (high-dentity lipoprotein-cholesterol [HDL]) [29]. Serum ox-LDL was measured using a commercial enzyme-linked immunosorbent assay (ELISA) kit for rats (Cusabio Biotech Co., Ltd., Wuhan, China) according to the manufacturer’s instructions [30]. Levels of ROM were determined using a free radical evaluator (Diacron International, Grosseto, Italy) according to previously reported analysis procedures [30]. Data are given in terms of Carratelli Units (CARR U), with 1 CARR U corresponding to 0.08 mg/dL hydrogen peroxide. Levels of serum CRP were also determined using a highly sensitive ELISA kit (Life Diagnostics, West Chester, PA) [31].

The measurements described above were performed in duplicate. Both intra- and inter-assay coefficients of variation were <5%.

### Determination of gene expression with real-time reverse transcription–polymerase chain reaction (RT-PCR)

The descending aorta was harvested, immediately frozen and kept in RNAlater, an RNA stabilization solution (Ambion, Austin, TX) at −80°C until use for real-time RT-PCR [26]. Total RNA was isolated from samples using Trizol reagent (Invitrogen, Carlsbad, CA) in accordance with the manufacturer’s instructions. Isolated RNA was quantified by measurement of absorbance at 260 nm, and purity was determined by the 260/280 nm absorbance ratio. Samples with a ratio >1.8 were used. Total RNA (2 μg) was reverse transcribed with Avian Myeloblastosis Virus Reverse Transcriptase (Takara, Shiga, Japan) at 42°C for 30 min. Prepared cDNA was diluted 10-fold with yeast RNA (10 μg/mL). Real-time PCR was performed using TOYOBO SYBR Green PCR Master Mix (Toyobo, Osaka, Japan) and the Mx3000P Real-time QPCR System (Agilent Technologies, Tokyo, Japan). Amplification conditions were as follows: 45 cycles at 95°C (5 s), 65°C (5 s) and 72°C (8 s) for *VCAM1;* and 45 cycles at 95°C (15 s), 59°C (20 s) and 72°C (20 s) for *TLR4*. Primer sequences were *VCAM1*, 5′-GGCTCGTACACCATCCGC-3′ and 5′-CGGTTTTCGATTCACACTCGT-3′ [32], and *TLR4*, 5′-GTGAGCATTGATGAGTTCAG-3′ and 5′-CATCTAATGATTGATAAGGATT-3′ [29]. Primers used to detect the internal control, β-actin, were 5′-TGTTGCCCTAGACTTCGAGCA-3′ and 5′-GGACCCAGGAAGGAAGGCT-3′ [29]. Expression of each gene is shown as the relative copy number ratio of the target gene against β-actin for each sample [29].

### Histological evaluation of aorta samples

Frozen sections (8 μm) were obtained from the descending aorta, embedded in Optimal Cutting Temperature compound (Tissue Tec; Miles, Naperville, IL) and stained with oil red O, hematoxylin and eosin, as described elsewhere [32]. Immunohistochemical staining for VCAM1 or ICAM1 was performed using Histofine Simple Stain MAX PO kits (Nichirei, Tokyo, Japan) [32]. Briefly, aortic tissues were immersed in methanol containing 0.3% hydrogen peroxide for 30 min in order to block endogenous peroxidase activity. Sections were then treated at 4°C with an anti-VCAM1 (Santa Cruz Biotechnology) [32] (diluted 1:100) or ICAM1 (diluted 1:50) overnight, followed by treatment with a secondary antibody (Fab’) with peroxidase complex for 30 min. Color was developed with a solution of 3,3-diaminobenzidine in 50 mmol/l Tris–HCl buffer (pH 7.5) containing 0.001% hydrogen peroxide and sections were counterstained with Mayer’s hematoxylin. Control sections included buffer alone or nonspecific purified rabbit immunoglobulin G. Percentages of total aortic lumen area occupied by plaques per section, the total aortic lumen area occupied by lipids per section, and VCAM1- or ICAM1-positive aortic lumen area were calculated using computer-assisted image analysis software (WinROOF, Mitani Co., Fukui, Japan) [32].

### Statistical analysis

All data analyses were performed using a statistical software package (PASW Statistics ver. 18.0; IBM Co., Tokyo, Japan). *T*-test was used for statistical comparison of the data between the control group and occlusal disharmony group. The level of significance was set at *p* < 0.05.

## Results

No significant differences were observed in food consumption between the two groups of rats over the experimental period. Body weights (mean ± SD) for the control and occlusal disharmony groups were 421.4 ± 29.1 and 410.0 ± 28.9 g, respectively, at 8 weeks. Body weights were not significantly different among the groups (*p* > 0.05).

Plasma corticosterone levels in the occlusal disharmony group were significantly higher than in the control group (1.3 times) (*p* < 0.05) (Table 1). Serum VLDL and LDL levels in the occlusal disharmony group were significantly higher than those in the control group (1.9 and 1.3 times, respectively) (*p* < 0.05) (Table 1). On the other hand, there were no significant differences in serum levels of oxidized LDL, ROM and CRP between the two groups (*p* > 0.05) (Table 1).

**Table 1:** **Difference in plasma/serum markers between control and occlusal disharmony groups**

Parameter	Control	Occlusal disharmony
Corticosterone (ng/mL)	14.2 ± 4.9^a^	28.6 ± 12.4^b^
Total cholesterol (mg/dL)	122.5 ± 11.1	162.8 ± 30.8^b^
Triglyceride (mg/dL)	48.5 ± 12.4	47.8 ± 24.5
Very low-density lipoprotein (mg/dL)	51.4 ± 6.1	66.1 ± 13.5^b^
Low-density lipoprotein (mg/dL)	47.1 ± 5.5	63.0 ± 11.6^b^
High-density lipoprotein (mg/dL)	9.8 ± 1.3	11.0 ± 1.7
Oxidized low density lipoprotein (ng/mL)	29.4 ± 5.0	25.8 ± 3.1
Reactive oxygen metabolites (CARR U)	469.3 ± 22.2	589.9 ± 163.0
C-reactive protein (mg/L)	1.0 ± 0.1	1.0 ± 0.2

^a^Data are expressed as means ± SD (n = 7).

^b^ *p* < 0.05 (vs. control group, according to *t*-test).

**Figure 1:**
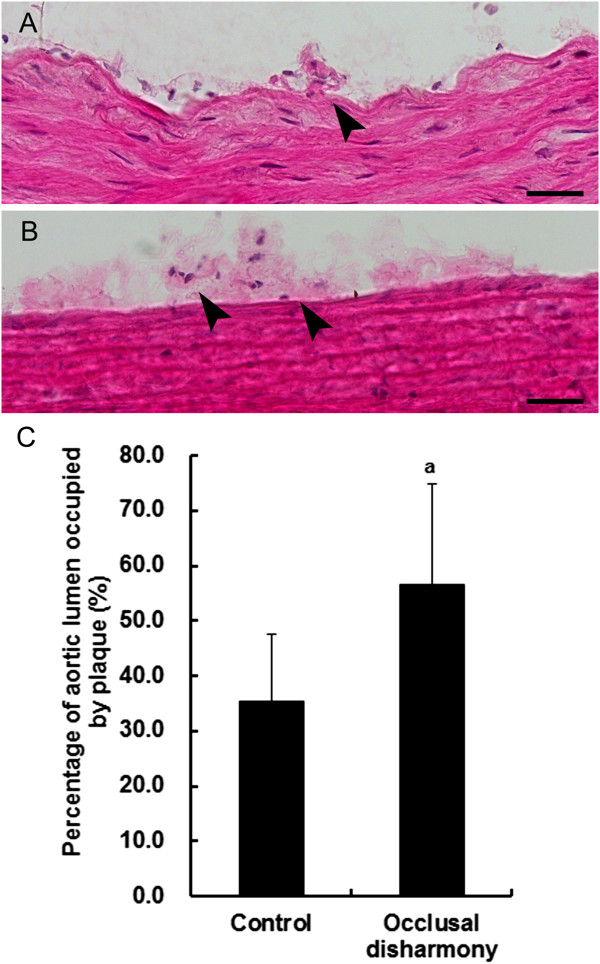
**Representative results of initial stages of atherosclerosis in descending aorta.** Macrophage collection (arrowheads) was observed in the control group **(A)** and occlusal disharmony groups **(B)**. Scale bar = 25 μm. Percentage of total aortic lumen area occupied by plaques (mean ± SD) was significantly higher in the occlusal disharmony group than in the control group **(C)**. ^a^ *p* < 0.05, vs. control group, according to *t*-test (n = 7).

**Figure 2:**
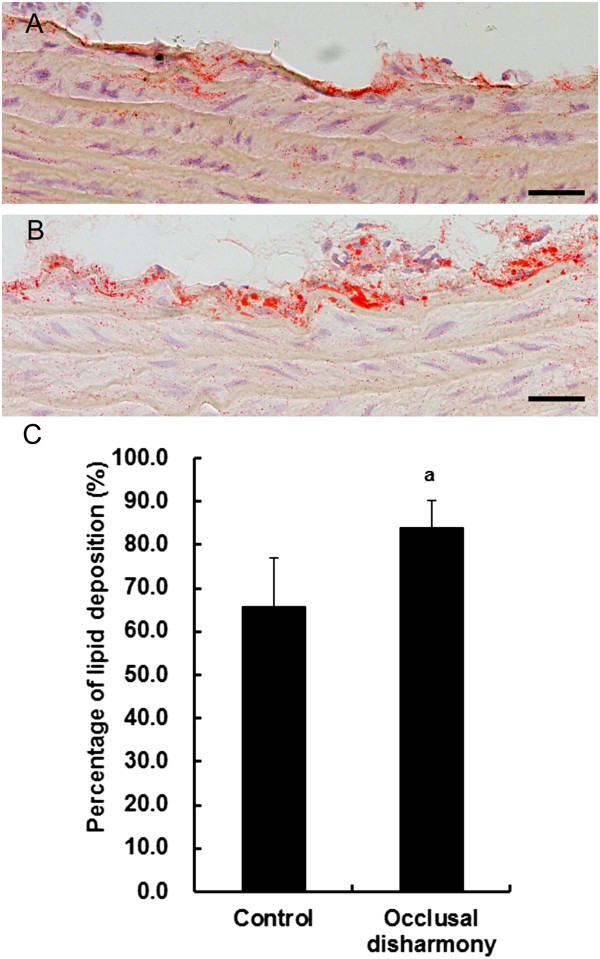
**Lipid deposition in descending aorta.** Lipid deposition (red color) in the occlusal disharmony group **(B)** was more intense than in the control group **(A)**. Scale bar = 25 μm. Area of lipid deposition in the occlusal disharmony group (mean ± SD) was significantly higher than in the control group **(C)**. ^a^ *p* < 0.05, vs. control group, according to *t*-test (n = 7).

**Figure 3:**
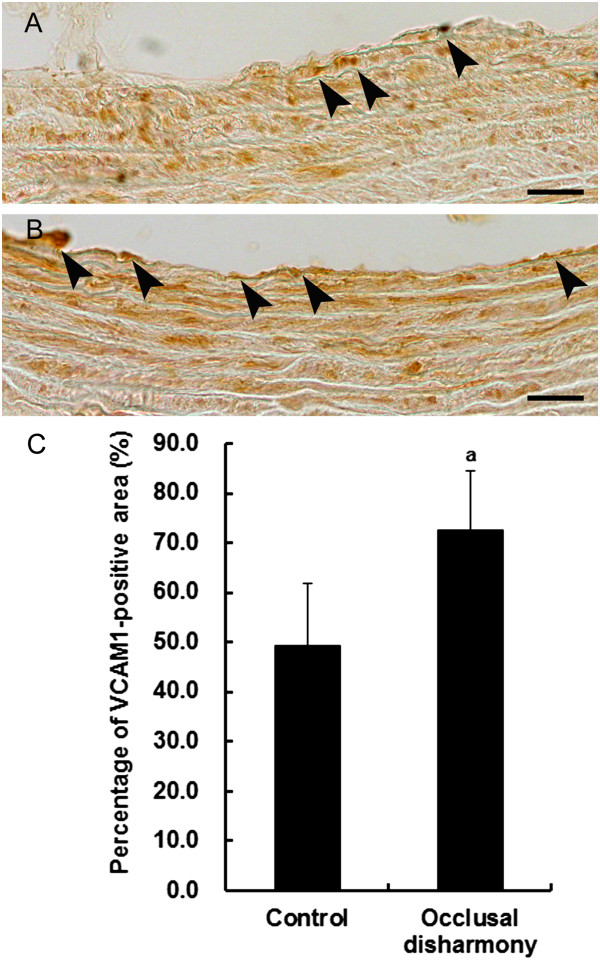
**Vascular cell adhesion molecule-1 (VCAM1) expression in cross sections of descending aorta.** VCAM1 expression in endothelial tissue (arrowheads) in the occlusal disharmony group **(B)** was more intense than in the control group **(A)**. Scale bar = 25 μm. Percentage of VCAM1-positive lumen (mean ± SD) in the occlusal disharmony group was significantly higher than that in the control group **(C)**. ^a^ *p* < 0.05, vs. control group, according to *t*-test (n = 7).

**Figure 4:**
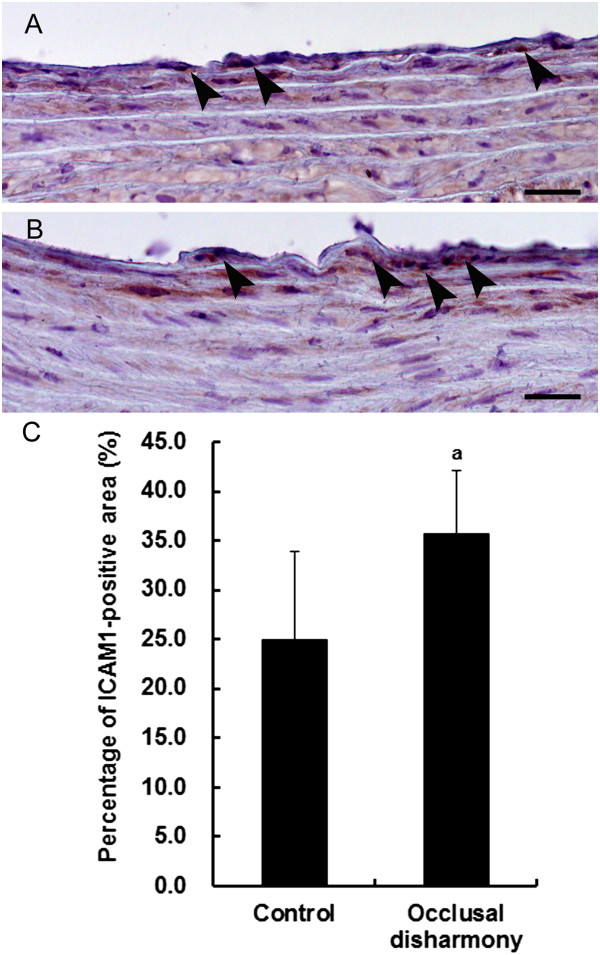
**Intracellular adhesion molecule-1 (ICAM1) expression in cross sections of descending aorta.** ICAM1 expression in endothelial tissue (arrowheads) in the occlusal disharmony group **(B)** was more intense than in the control group **(A)**. Scale bar = 25 μm. Percentage of ICAM1-positive lumen (mean ± SD) in the occlusal disharmony group was significantly higher than that in the control group **(C)**. ^a^ *p* < 0.05, vs. control group, according to *t*-test (n = 7).

The percentage of total aortic lumen area occupied by plaques was significantly higher in the occlusal disharmony group than that in the control group (*p* < 0.05), although the plaque sizes were small and only early lesions of atherosclerosis were observed (Figure 1). The area of lipid deposition in the occlusal disharmony group was significantly higher than that in the control group (Figure 2). The VCAM1-positive area was significantly higher in the occlusal disharmony group than in the control group (1.5 times) (*p* < 0.05) (Figure 3). The ICAM1-positive area was significantly higher in the occlusal disharmony group than in the control group (1.4 times) (*p* < 0.05) (Figure 4).

Gene expression of *VCAM1* and *TLR4* was significantly higher in the occlusal disharmony group than in the control group (1.9 and 4.3 times, respectively) (*p* < 0.05) (Table 2).

**Table 2:** **Fold change in gene expression in rat descending aorta**

Parameter	Control	Occlusal disharmony
*Vascular cell adhesion molecule-1*	0.41 ± 0.07^a^	0.79 ± 0.05^b^
*Toll-like receptor 4*	0.32 ± 0.08	1.36 ± 0.21^b^

^a^Data are expressed as means ± SD (n = 3 independent experiments). mRNA levels were calculated in terms of relative copy number ratio for each mRNA against β-actin for each sample.

^b^ *p* < 0.05 (vs. control group, according to *t*-test).

## Discussion

Psychosocial stress, particularly chronic stress, is a nontraditional risk factor for atherosclerosis in humans [17, 18]. Experimental studies have also demonstrated that chronic stress accelerates atherosclerosis [19, 20], and occlusal disharmony induces psychological stress [21–24]. However, the mechanisms by which occlusal disharmony accelerates the initiation of atherosclerosis are not clearly understood. To the best of our knowledge, this is the first study to assess the causal relationship between occlusal disharmony and initiation of atherosclerosis in the rat descending aorta. In this study, the occlusal disharmony group showed higher plasma levels of corticosterone than the control group. Furthermore, in the occlusal disharmony group, the percentages of total aortic lumen area occupied by plaques and lipid deposition were significantly higher than those in the control group. These findings suggest that psychological stress induced by occlusal disharmony accelerated atherosclerosis.

TLR4 plays an important role in the initiation of atherosclerosis [4]. Increased expression of TLR4 within lipid-rich atherosclerotic plaques in both human and animal models has been reported [33, 34]. While TLR4 was originally described as a pattern receptor that recognizes lipopolysaccharide, endogenous ligands, such as ox-LDL, fibronectin and heat shock protein, are known to be TLR4 activators [35]. Modifications of LDL, icluding ceramide-enriched LDL can induce TLR4 expression [36]. In this study, serum levels of LDL and aortic *TLR4* gene expression in the occlusal disharmony were significantly higher than those in the control group. TLR4 expression following occlusal disharmony may be up-regulated by LDL.

VCAM1 and ICAM1 are endothelial adhesion molecules of the Ig gene superfamily that may participate in atherogenesis by promoting monocyte accumulation in the arterial intima [37]. Although expression of both VCAM-1 and ICAM-1 is up-regulated in atherosclerotic lesions, VCAM1, but not ICAM1, plays a dominant role in the initiation of atherosclerosis [38]. On the other hand, LDL induces expression of VCAM1 in human vascular endothelial cells [39]. In this study, the protein and gene expression of VCAM1 in the aorta, the area of lipid deposition, and the serum levels for LDL were significantly higher in the occlusal disharmony group when compared to the control group. These findings suggest that LDL induced by occlusal disharmony induces VCAM1 expression and accelerates the initiation of atherosclerosis.

Although it remains unclear how corticosterone induced by occlusal disharmony contributes to up-regulation of total cholesterol or LDL in this study, there may be a possible relationship between corticosterone and regulation of lipid and lipoprotein metabolism. Exposure to low levels of corticosterone for long terms significantly exacerbates atherosclerosis in ApoE knockout mice [40]. Synthetic glucocorticoid treatment increases the expression of the enzyme 3-hydroxy-3-methyl-glutaryl-CoA reductase (HMG-CoA reductase) [41]. HMG-CoA reductase is a key regulatory enzyme in the conversion of HMG-CoA to mevalonic acid, which is a precursor for cholesterol synthesis [42]. Competitive inhibitors of HMG-CoA reductase increase the expression of LDL receptors in the liver, which decreases serum total cholesterol and LDL concentrations [43]. Taken together, these findings suggest that corticosterone exacerbates atherosclerosis by primarily up-regulating circulating levels of total cholesterol and LDL [40].

There were no significant differences in serum levels of ox-LDL and ROM between the control and occlusal disharmony groups. An increasing number of studies have demonstrated that oxidative stress plays a pivotal role in the pathogenesis of atherosclerosis [17, 44]. ROS peroxidizes lipid components, leading to the formation of ox-LDL, which plays a role in the development and progression of atherosclerosis and its complications [45]. Ox-LDL is formed by oxidative stress and leads to endothelial activation and injury resulting in an inflammatory response that induces recruitment, activation and migration of monocytes through inter-endothelial gaps to the sub-endothelial region [46]. These observations have been confirmed in stress models, in which restraint stress up-regulates lectin-like ox-LDL receptor-1 via formation of ROS in the aorta of apolipoprotein E-deficient mice [47]. However, our findings differed from the results of a previous study. The reasons for this discrepancy are not clear, but may depend on the type of psychological stress (restraint stress vs. occlusal disharmony) and species (mouse vs. rat).

A chronic inflammatory response contributes to atherogenesis and plays a critical role in the initiation of this process [14]. CRP is a marker of inflammation and is a strong marker for cardiovascular morbidity [48]. In this study, there were no significant differences in serum levels of CRP between the control and occlusal disharmony groups. However, in a previous study, chronic unpredictable stress accelerated atherosclerosis by increasing serum levels of CRP in apolipoprotein E knockout mice [20]. This chronic unpredictable stress included heat stimulation, cage tilting, wet bedding, lights on overnight, tail pinch, high-speed agitation, cold stimulation, overhang, water deprivation and food deprivation. These conditions significantly reduced body weight and induced 2.5-fold higher levels of aortic VCAM1 expression [20]. The psychological stress induced by occlusal disharmony in our study is milder than the unpredictable stress in the previous study. In fact, there was no body weight loss in our stress model.

Psychosocial stress is one of the risk factors for atherosclerosis in humans [17, 18]. Although occlusal disharmony induces psychological stress in animal [22–24] and in human [21] models, the relationship between psychological stress from occlusal disharmony and atherosclerosis is not clear in humans. Recently, cross-sectional epidemiological studies have reported on the relationship between tooth loss and atherosclerosis [49, 50]. Number of teeth was related to atherosclerotic plaque in the carotid arteries in an elderly Swedish population [49]. A positive relationship between increased tooth loss in the posterior region and accumulation of arterial atheroma has been shown in a Korean population [50]. These results suggest that occlusal disharmony from tooth loss induces psychological stress and is a risk factor for atherosclerosis in humans. Thus, improvement of occlusal disharmony, such as through prosthodontic therapy, may contribute to the prevention and treatment of atherosclerosis. However, further studies are necessary to clarify the relationship between improved occlusal disharmony and atherosclerosis.

Our study has several limitations. In this model, only the initial stage of atherosclerosis was observed, rather than advanced lesions. Although our occlusal disharmony model that induces psychological stress was established for an 8-week experimental period [24], longer-term follow-up may provide additional information on the development of atherosclerosis. Furthermore, there is no data whether atherosclerosis is diminished by suppressing the plasma corticosterone level in our occlusal disharmony rats.

In conclusion, in the apoE knockout rats, occlusal disharmony may induce VCAM1, ICAM1 and TLR4 expression and accelerate the initiation of atherosclerosis.

## Abbreviations

VCAM1: Vascular cell adhesion molecule-1
ICAM1: Intercellular adhesion molecule-1
LDL: Low-density lipoprotein
TLR4: Toll-like receptor 4
ROS: Reactive oxygen species
ox-LDL: Oxidized LDL
apoE: Apolipoprotein E
ROM: Reactive oxygen metabolites
CRP: C-reactive protein
VLDL: Very low-density lipoprotein
HDL: High-dentity lipoprotein
ELISA: Enzyme-linked immunosorbent assay
RT-PCR: Reverse transcription–polymerase chain reaction
CARR U: Carratelli Unit
HMG-CoA reductase: 3-hydroxy-3-methyl-glutaryl-CoA reductase.
